# Periodontal Inflammatory Burden and Multi-Organ Microvascular Impairment in Type 2 Diabetes: A Cross-Sectional Observational Study

**DOI:** 10.3390/cimb48060601

**Published:** 2026-06-05

**Authors:** Maria-Alexandra Martu, Stefan-Lucian Burlea, Silvia Martu, Sorina-Mihaela Solomon, Ionut Luchian, Liliana Pasarin, Ioana Martu, Mihaela Salceanu, Elena-Odette Luca, Diana-Maria Anton, Diana Tatarciuc, Irina-Georgeta Sufaru

**Affiliations:** Grigore T. Popa University of Medicine and Pharmacy, 700115 Iasi, Romania; maria-alexandra.martu@umfiasi.ro (M.-A.M.); sorina.solomon@umfiasi.ro (S.-M.S.); liliana.pasarin@umfiasi.ro (L.P.); ioana.martu@umfiasi.ro (I.M.); mihaela.salceanu@umfiasi.ro (M.S.); elena-odette.luca@umfiasi.ro (E.-O.L.); diana_maria_a@yahoo.com (D.-M.A.); diana.tatarciuc@umfiasi.ro (D.T.); ursarescu.irina@umfiasi.ro (I.-G.S.)

**Keywords:** periodontitis, periodontal inflamed surface area (PISA), type 2 diabetes, OCT angiography, microvascular dysfunction, endothelial activation

## Abstract

Periodontitis and type 2 diabetes mellitus (T2DM) are linked through systemic inflammation and endothelial dysfunction, yet it remains uncertain whether periodontal inflammatory burden independently reflects early, multi-organ microvascular vulnerability beyond glycemic exposure. This study aimed to assess the independent association between periodontal inflammatory burden, measured by PISA, and retinal microvascular impairment on OCT-A, and to examine relationships with renal trajectories, small-fiber neuropathy, and inflammatory/endothelial biomarkers. This cross-sectional observational study included 285 never-smoking adults with T2DM. The primary outcome was a pre-specified OCT-A microvascular impairment composite. Secondary outcomes included eGFR slope and log(UACR) slope, corneal nerve fiber length (CNFL), and a multi-organ microvascular burden score. Biomarkers comprised hsCRP, IL-6, sICAM-1, sVCAM-1, sE-selectin, PAI-1, angiopoietin-2 (Ang-2), and vWF:Ag. Multivariable linear regression estimated associations per 1 SD higher PISA, adjusting for age, sex, diabetes duration, HbA1c, CGM time in range, CGM coefficient of variation, systolic blood pressure, LDL cholesterol, BMI, and medication classes (SGLT2 inhibitors, GLP-1 receptor agonists, ACEi/ARB, statins). False discovery rate (FDR) control (*q* = 0.10) was applied for secondary endpoints. Higher PISA was independently associated with worse OCT-A microvascular impairment (adjusted *β* = 0.138, 95% CI 0.061–0.216; *p* = 0.0005). Although statistically significant, the effect sizes were modest in magnitude, and their translation into clinically meaningful differences in microvascular outcomes warrants investigation in prospective settings. Higher PISA was also associated with greater multi-organ microvascular burden (*β* = 0.101, 95% CI 0.040–0.163; *p* = 0.0014; FDR *q* = 0.005) and lower CNFL (*β* = −0.224, 95% CI −0.397 to −0.052; *p* = 0.0113; FDR *q* = 0.023). PISA was associated with higher levels of inflammatory and endothelial activation/injury biomarkers (all FDR *q* < 0.10). In this cross-sectional study, periodontal inflammatory burden was independently associated with quantitative retinal microvascular impairment, lower corneal nerve fiber length, and a consistent pattern of endothelial activation biomarker elevations in never-smoking adults with T2DM. The clinical significance of the observed effect sizes requires further evaluation, and longitudinal studies are needed to establish temporality.

## 1. Introduction

Type 2 diabetes mellitus (T2DM) remains a leading cause of preventable vision loss, chronic kidney disease, and neuropathy, largely through progressive microvascular injury [1]. Although contemporary care has improved glycemic control and cardiovascular risk management [2], substantial residual risk persists, and many patients develop early microvascular dysfunction before conventional clinical endpoints are detectable [3]. Sensitive, quantitative phenotypes that capture preclinical retinal, renal, and neural microangiopathy are therefore increasingly used to refine risk stratification and to identify mechanistic pathways amenable to integrated prevention strategies [4,5].

Periodontitis is a prevalent chronic inflammatory disease characterized by a dysbiotic subgingival biofilm and a host immune-inflammatory response that produces ulcerated pocket epithelium and systemic dissemination of microbial products and inflammatory mediators [6,7]. The bidirectional relationship between diabetes and periodontitis is well recognized, with hyperglycemia worsening periodontal susceptibility and periodontal inflammation potentially contributing to metabolic dysfunction via systemic inflammation and endothelial perturbation [7,8,9,10,11]. However, beyond glycemic control, it remains uncertain whether the periodontal inflammatory burden independently signals microvascular vulnerability in T2DM and, if so, which microvascular beds are most strongly linked to oral inflammation.

Existing observational evidence supports an association between periodontitis and diabetic retinopathy (DR) [12,13,14], with a recent meta-analysis confirming higher odds of DR among people with diabetes and periodontitis, while noting heterogeneity in periodontal and retinopathy phenotyping [14]. However, most studies have relied on binary DR classifications that may miss early perfusion abnormalities. Optical coherence tomography angiography (OCT-A) enables quantification of capillary non-perfusion, vessel density, and foveal avascular zone metrics before clinically apparent DR [15], and its sensitivity to glycemic exposure in T2DM without clinical retinopathy highlights the value of integrating continuous glucose monitoring (CGM) metrics when modeling microvascular outcomes [16].

Renal and neural microvascular complications provide complementary windows into systemic microangiopathy. Baseline periodontitis severity and PISA have been prospectively associated with subsequent eGFR decline in T2DM, with effects not fully mediated by glycemic control [17]. For neuropathy, corneal confocal microscopy (CCM) has emerged as a sensitive method for quantifying small-fiber loss, with longitudinal data showing that sustained corneal nerve damage predicts the incidence of diabetic peripheral neuropathy [18]. Together, these modalities enable multi-organ, quantitative assessment of microvascular health beyond conventional complication staging. Constructing a composite multi-organ microvascular burden score from these complementary assessments is conceptually grounded in the understanding that diabetic microangiopathy is a systemic process affecting multiple vascular beds through shared pathophysiological mechanisms, including chronic inflammation, endothelial dysfunction, and impaired perfusion [19]. A composite score captures the cumulative microvascular impact that single-organ measures may underestimate and reduces multiplicity concerns inherent in analyzing each organ system separately.

A mechanistic link between periodontal inflammation and microvascular disease is biologically plausible and may involve systemic inflammation, endothelial activation, and prothrombotic imbalance. Reviews of diabetic vascular disease emphasize that endothelial dysfunction is central to microvascular complications, driven by inflammation, oxidative stress, and impaired nitric oxide bioavailability [19]. Circulating adhesion molecules and selectins reflect endothelial activation and leukocyte–endothelium interactions; in a follow-up study, baseline levels of VCAM-1 and selectins were associated with subsequent development of diabetic neuropathy, retinopathy, and nephropathy [20]. PISA is particularly relevant in this context because it quantifies the surface area of inflamed, bleeding periodontal pocket epithelium, providing a continuous measure of periodontal inflammatory burden rather than categorical disease definitions [21]. Recent work also supports links between higher PISA and systemic inflammatory biomarkers: in a clinico-hematological study, PISA showed a significant positive correlation with serum CRP (*r* = 0.49, *p* < 0.001) and ESR (*r* = 0.41, *p* < 0.001), confirming that the surface area of inflamed pocket epithelium tracks with circulating inflammatory markers [22,23]. These findings reinforce the utility of PISA as a biologically grounded exposure in cardio-metabolic research. While this mechanistic framework provides biological plausibility for an association between periodontal inflammation and microvascular outcomes, it should be noted that the present cross-sectional design cannot establish directional biological pathways, and the mechanistic links outlined above remain hypothetical until confirmed by interventional or longitudinal evidence.

Against this background, the present observational study was designed to test whether periodontal inflammatory burden, quantified by PISA, is independently associated with high-impact microvascular phenotypes in never-smoking adults with T2DM. By combining OCT-A retinal perfusion metrics with kidney trajectory measures based on albuminuria and eGFR slopes, CCM-based small-fiber outcomes, and a focused biomarker panel reflecting systemic inflammation and endothelial activation/injury, the study aims to clarify whether periodontitis is associated with a clinically measurable microvascular vulnerability phenotype beyond glycemic exposure and conventional cardiometabolic risk factors.

The primary aim was to evaluate the independent association between PISA and a composite OCT-A microvascular impairment score after adjustment for demographic factors, diabetes duration, HbA1c, and CGM metrics, blood pressure, lipids, adiposity, and cardiometabolic medications. Secondary aims were to examine associations between PISA and renal trajectories (albuminuria and eGFR slopes), CCM-derived small-fiber metrics, a multi-organ microvascular burden score, and circulating biomarkers of systemic inflammation (hsCRP, IL-6) and endothelial activation/injury (sICAM-1, sVCAM-1, sE-selectin, PAI-1, vWF:Ag, and angiopoietin-2). Importantly, the cross-sectional design does not permit determination of directionality; the observed associations could reflect an influence of periodontal inflammation on microvascular outcomes, a greater susceptibility to periodontal inflammation among individuals with more advanced systemic microvascular disease, or shared underlying risk factors driving both conditions.

## 2. Materials and Methods

### 2.1. Study Design and Setting

This observational study used a deep-phenotyping, single-visit design (T0) in adults with type 2 diabetes mellitus (T2DM), complemented by retrospective extraction of renal biomarkers to model prior eGFR and urinary albumin-to-creatinine ratio (UACR) trajectories. The study was conducted at the Periodontology Clinic of Grigore T. Popa University of Medicine and Pharmacy in Romania, between September 2021 and July 2025. All procedures were performed in accordance with the Declaration of Helsinki. The Institutional Review Ethics Committee of Grigore T. Popa University of Medicine and Pharmacy in Iasi, Romania, approved the study (approval date: 30 July 2020), and all participants provided written informed consent.

### 2.2. Participants

Consecutive patients attending diabetes and allied clinics were screened for eligibility. Inclusion criteria were age 18 years of age or older, a documented diagnosis of T2DM, self-reported never-smoker status (defined as lifetime consumption <100 cigarettes and no use of other nicotine or tobacco products), availability of glycated hemoglobin (HbA1c) obtained within ±4 weeks of T0, availability of continuous glucose monitoring (CGM) data for a standardized analysis window, and availability of historical renal data enabling estimation of eGFR slope and UACR trajectory. Participants were excluded if they had type 1 or secondary diabetes, pregnancy, acute infection or hospitalization within the previous 3 months, systemic inflammatory or autoimmune diseases likely to confound the assessment of inflammatory burden, chronic use of systemic immunosuppressants, ocular conditions precluding reliable OCT angiography acquisition, or recent ocular surgery within 3 months.

### 2.3. Clinical and Metabolic Characterization

At T0, demographic and clinical data were collected using standardized case report forms and, where applicable, verified against medical records. Recorded variables included age, sex, diabetes duration, antihyperglycemic therapy (including SGLT2 inhibitors and GLP-1 receptor agonists), antihypertensive therapy (including ACE inhibitors/angiotensin receptor blockers), statin therapy, body mass index (BMI), and blood pressure (mean of two seated measurements after at least 5 min of rest). Macrovascular disease history was recorded. Socioeconomic status proxies and oral hygiene behaviors were not systematically captured and therefore were not available for inclusion in the adjusted models.

### 2.4. Periodontal Examination and Periodontal Inflammatory Burden

All periodontal examinations were performed at T0 by calibrated dental examiners blinded to ocular and neuropathy measurements. Examiner calibration was performed before study initiation on non-study patients; inter-examiner agreement for probing pocket depth (PPD) and clinical attachment level (CAL) was assessed using intraclass correlation coefficients, with a pre-defined target of at least 0.80.

Full-mouth periodontal charting was conducted at six sites per tooth (excluding third molars) using a CE-marked manual periodontal probe (UNC-15 or equivalent). PPD and CAL were recorded in millimeters; bleeding on probing (BOP) was recorded as present/absent within 15 s after probing, and plaque index was recorded when feasible. Periodontitis was classified using the 2017 World Workshop staging and grading framework [24].

The primary periodontal exposure was periodontal inflamed surface area (PISA), expressed in mm^2^, calculated from site-level PPD/CAL measurements and BOP status using established algorithms to estimate the surface area of inflamed pocket epithelium, then summed across all teeth. Secondary periodontal exposures included BOP percentage, mean PPD, number of sites with PPD ≥6 mm, tooth loss count, and periodontitis Stage and Grade.

### 2.5. Ocular Microvascular Assessment (OCT Angiography)

Retinal microvascular structure was assessed at T0 using optical coherence tomography angiography (OCT-A) on the ZEISS CIRRUS 6000 OCT platform with AngioPlex (Carl Zeiss Meditec AG, Jena, Germany), a system routinely used in Romanian ophthalmology practice. Imaging was performed by trained technicians blinded to periodontal findings, in accordance with local clinical protocols for pupil dilation. The device software version and the angiography analysis package used at acquisition were recorded and kept constant for primary analyses.

Macular OCT-A scans were acquired with a prespecified 3 × 3 mm macular scan size for the primary analyses; a 6 × 6 mm scan was also collected when feasible for exploratory analyses. Both eyes were imaged, and the eye with higher overall image quality (signal strength and artifact-free) was designated as the study eye; if the quality was equivalent, the right eye was selected. Scans were accepted if they met the manufacturer-recommended image quality threshold (signal strength index ≥7) and showed no major motion artifacts or clinically relevant segmentation errors; otherwise, imaging was repeated during the same visit when possible. Automated layer segmentation was reviewed and corrected in accordance with standard clinical practice before metric export.

To minimize multiplicity, a single primary OCT-A outcome was pre-specified as a retinal microvascular impairment composite score. The composite was constructed from three study-eye OCT-A metrics available for all participants: superficial capillary plexus vessel density (SCP-VD, %), deep capillary plexus vessel density (DCP-VD, %), and foveal avascular zone area (FAZ, mm^2^). These metrics were selected a priori because they reflect complementary aspects of retinal capillary perfusion and macular microvascular integrity. Each metric was standardized as a z-score using the study sample mean and standard deviation. Directions were aligned so that higher values indicated worse retinal microvascular status; thus, SCP-VD and DCP-VD were reverse-coded before standardization, whereas FAZ area was retained in its original direction. The composite was then calculated as the arithmetic mean of the three aligned z-scores:

$$\mathrm{OCT-A}\;\mathrm{composite}=\frac{z\left(FAZ\;area\right)\;-\;z\left(\mathrm{SCP-VD}\right)\;-\;z(\mathrm{DCP-VD})}{3}$$

Higher composite values, therefore, indicate greater retinal microvascular impairment. Equal weighting was used to maximize transparency and reproducibility, and because no strong prior evidence supported differential weighting of the three components. Secondary ocular outcomes included the individual OCT-A components and, when available, the clinical diabetic retinopathy stage.

### 2.6. Neuropathy Phenotyping (Corneal Confocal Microscopy)

Small-fiber neuropathy was phenotyped at T0 using in vivo corneal confocal microscopy with the Heidelberg Retina Tomograph 3 equipped with the Rostock Cornea Module (HRT3-RCM; Heidelberg Engineering GmbH, Heidelberg, Germany). After topical anesthesia (e.g., oxybuprocaine 0.4%), high-quality images of the central corneal subbasal nerve plexus were acquired from both eyes using a standardized protocol. Images were analyzed using validated automated or semi-automated software; the software name and version were recorded and kept constant for primary analyses. The pre-specified primary CCM endpoint was corneal nerve fiber length (CNFL, mm/mm^2^), with corneal nerve fiber density (CNFD) and corneal nerve branch density (CNBD) analyzed as secondary endpoints. If CCM could not be obtained in a small subset of participants due to technical or ocular limitations, quantitative sensory testing was performed as a predefined alternative neuropathy phenotype and analyzed in sensitivity analyses.

### 2.7. Renal Outcomes and Trajectories (Retrospective Extraction)

Renal laboratory data were extracted from electronic medical records and/or laboratory information systems for the 24 to 36 months preceding T0. Serum creatinine values were used to compute eGFR with a single pre-specified equation applied consistently across the dataset (CKD-EPI 2021 or the locally adopted CKD-EPI implementation; the chosen equation was pre-specified in the analysis plan). UACR values from spot urine samples were collected and expressed as mg/g; if reported in mg/mmol, values were converted using documented factors.

eGFR slope (mL/min/1.73 m^2^ per year) was estimated for each participant using ordinary least-squares regression of eGFR on time (in years), requiring at least 3 measurements spanning at least 12 months, with a preference for at least 24 months when available. In sensitivity analyses, mixed-effects models with random intercepts and slopes were used to account for unequal measurement timing and within-subject variability. The UACR trajectory was modeled using natural log-transformed UACR due to right skew; a slope was estimated when at least 2 UACR measurements were available. As a clinically interpretable alternative, albuminuria progression categories were defined using standard thresholds (normoalbuminuria, moderately increased albuminuria, severely increased albuminuria).

### 2.8. Glycemic Exposure (CGM and HbA1c)

HbA1c was measured in an accredited laboratory within ±4 weeks of T0. CGM data were obtained from participants’ routine devices available in Romania, including Dexcom ONE+ (Dexcom, Inc., San Diego, CA, USA) and FreeStyle Libre 2 systems (Abbott Diabetes Care). CGM reports and raw data were exported using manufacturer platforms (e.g., Dexcom Clarity for Dexcom systems and LibreView for FreeStyle Libre systems). To standardize exposure, CGM metrics were calculated over a pre-specified window of at least 10 consecutive days with at least 70% data capture. The pre-specified CGM metrics were time in range (70–180 mg/dL, %), time above range (>180 mg/dL, %), coefficient of variation (CV, %), and mean glucose.

### 2.9. Inflammatory and Endothelial Biomarkers

At T0, venous blood was collected for assessment of inflammatory and endothelial biomarkers. Serum was used for high-sensitivity C-reactive protein (hsCRP), while EDTA plasma was used for cytokine and endothelial biomarkers. Samples were processed within 2 h of collection, centrifuged at approximately 1500–2000× *g* for 10–15 min at 4 °C, aliquoted, and stored at −80 °C until batch analysis; repeated freeze–thaw cycles were avoided.

hsCRP was quantified using a high-sensitivity immunoturbidimetric method on an automated clinical chemistry analyzer in an accredited Romanian clinical laboratory, according to the manufacturer’s instructions and routine internal/external quality assurance procedures.

Endothelial activation biomarkers (soluble intercellular adhesion molecule-1 [sICAM-1], soluble vascular cell adhesion molecule-1 [sVCAM-1], soluble E-selectin [sE-selectin], and plasminogen activator inhibitor-1 [PAI-1]) together with a glycocalyx-related marker (angiopoietin-2 [Ang-2]) and interleukin-6 [IL-6], were measured using a multiplex electrochemiluminescence immunoassay platform (Meso Scale Discovery, Rockville, MD, USA). Assay kit names, catalog numbers, and lot numbers were recorded, and all samples were analyzed in duplicate using the same platform and kit lots when feasible. Inter- and intra-assay coefficients of variation provided by the manufacturer and observed in study quality-control samples were documented.

Endothelial injury was assessed using von Willebrand factor antigen (vWF:Ag), measured by immunoassay according to the local laboratory’s standardized protocol (e.g., ELISA or automated immunoassay), with calibration and quality control performed per manufacturer guidance. All biomarker assays were conducted by personnel blinded to periodontal status and imaging outcomes.

### 2.10. Blinding and Data Management

Periodontal examiners were blinded to OCT-A and neuropathy outputs, and imaging technicians were blinded to periodontal status. Data were pseudonymized at entry and stored in a password-protected database. All enrolled participants had complete data for the pre-specified exposures, outcomes, and covariates used in the primary and secondary models; therefore, no imputation procedures were required.

### 2.11. Statistical Analysis

Analyses were performed using R (R 4.5.3, R Foundation for Statistical Computing, Vienna, Austria). Two-sided *p*-values < 0.05 were considered statistically significant for the primary hypothesis. The primary hypothesis tested whether a higher periodontal inflammatory burden (PISA, modeled per standard deviation increase) was associated with worse retinal microvascular status as captured by the OCT-A composite score.

The primary association was evaluated using multivariable linear regression with the OCT-A composite score as the dependent variable and PISA as the independent variable of interest. The pre-specified adjustment set included age, sex, diabetes duration, HbA1c, CGM time in range, CGM coefficient of variation, systolic blood pressure, LDL cholesterol, BMI, and medication classes (SGLT2 inhibitor use, GLP-1 receptor agonist use, ACE inhibitor/angiotensin receptor blocker use, and statin use). Secondary analyses evaluated associations of PISA with eGFR slope, log(UACR) slope, and CNFL using analogous multivariable models. A multi-organ microvascular burden score was constructed as the mean of standardized z-scores from the OCT-A composite, a renal trajectory metric (eGFR slope and/or UACR slope), and CNFL, and was analyzed as a secondary outcome. Biomarker concentrations were log-transformed when appropriate and standardized to z-scores; associations between PISA and biomarkers, and between biomarkers and microvascular outcomes, were examined in pre-specified secondary models, with multiplicity controlled using the same false discovery rate approach. The OCT-A composite was analyzed as a continuous, standardized outcome; positive regression coefficients indicate worse retinal microvascular status per each 1-SD increase in PISA.

To examine the internal coherence of the composite endpoint, separate multivariable linear regression models were also fitted for SCP-VD, DCP-VD, and FAZ area using the same adjustment set as in the primary analysis.

Effect modification by glycemic variability was examined by adding interaction terms between PISA and CGM-derived variability (CV) and between PISA and time in range to the primary model. To address multiplicity, only one primary endpoint (OCT-A composite) was tested at *α* = 0.05. Secondary endpoints were interpreted using the Benjamini–Hochberg false discovery rate procedure with *q* = 0.10.

Sensitivity analyses for the primary endpoint included nested adjustment models to assess the influence of glycemic covariate specification, HC3 robust standard errors, log-transformation of PISA, alternative periodontal exposure definitions (BOP%, mean PPD, and number of sites with PPD ≥6 mm), and exclusion of participants with advanced retinopathy or severely increased albuminuria to reduce reverse-causation concerns.

### 2.12. Power Analysis

Power was assessed as a minimum detectable effect (MDE) for the pre-specified primary association between periodontal inflammatory burden (PISA) and the OCT-A retinal microvascular composite, given the planned sample size (N = 285). The primary hypothesis test was the incremental contribution of PISA (1 df) in a multivariable linear regression model, evaluated with an F-test at *α* = 0.05 (two-sided). The a priori adjustment set included age; sex; diabetes duration; systolic blood pressure; LDL cholesterol; BMI; and use of SGLT2 inhibitors, GLP-1 receptor agonists, ACE inhibitor/angiotensin receptor blockers, and statins. Glycemic exposure was modeled parsimoniously to limit collinearity, using HbA1c together with two CGM-derived metrics capturing mean control and variability (time in range and coefficient of variation), yielding 12 covariates in the primary model and residual degrees of freedom df_2_ = 285 − 12 − 1 = 272. Under these assumptions, the study has 80% power to detect an incremental effect size for PISA of approximately Cohen’s *f^2^* ≈ 0.029, corresponding to a partial R^2^ ≈ 0.028 (partial correlation ≈ 0.17) for PISA after adjustment for covariates. Accordingly, the study is powered to detect small independent associations for the primary OCT-A endpoint; analyses involving secondary outcomes, interaction terms, and biomarker endpoints are considered secondary/exploratory and were not individually powered.

## 3. Results

### 3.1. Participant Characteristics

All 285 participants were included in the analyses, and the dataset was complete for all pre-specified exposures, outcomes, and covariates. Participants had a mean age of 60.8 ± 9.2 years, and 52.6% were men. Median PISA was 560 mm^2^ (IQR 316–827). Mean HbA1c was 7.11 ± 0.84%, with CGM time in range of 74.7 ± 10.3% and coefficient of variation of 32.8 ± 7.2%. Table 1 summarizes baseline characteristics overall and by PISA tertiles.

Periodontitis stage distribution differed markedly across PISA tertiles (chi-square *p* < 0.001), with Stage I observed only in the low-PISA tertile, Stage II predominating in the mid-PISA tertile, and Stages III–IV concentrated in the high-PISA tertile. In contrast, the distribution of periodontitis grades did not differ significantly across tertiles (chi-square *p* = 0.678), with Grade B being the most frequent category overall (Table S1).

### 3.2. Retinal Microvascular Outcomes

**Figure 1:**
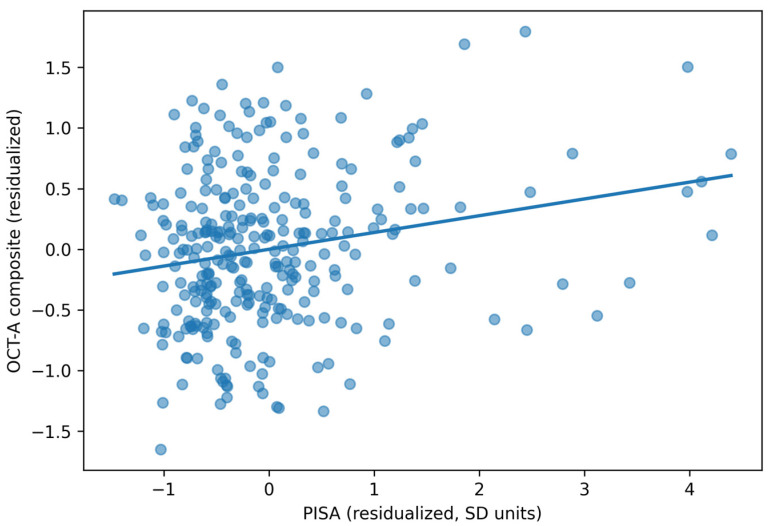
Adjusted association between PISA and the retinal OCT-A composite score (partial residual plot).

In the primary multivariable model, higher periodontal inflammatory burden was independently associated with worse retinal microvascular status, as reflected by a higher OCT-A composite score (indicating a larger FAZ area) and lower superficial and deep capillary plexus vessel density. Each 1 SD higher PISA was associated with a 0.138 SD higher OCT-A microvascular impairment composite score (95% CI 0.061 to 0.216; *p* = 0.0005). The fully adjusted model explained 53.7% of the variance in the OCT-A composite (*R^2^* = 0.537; adjusted *R^2^* = 0.513). The adjusted association is shown in Figure 1.

There was no evidence of effect modification by CGM-derived glycemic variability or time in range in the fully adjusted interaction models (PISA×CV interaction *p* = 0.7401; PISA×TIR interaction *p* = 0.6151).

To ensure that the composite result was clinically interpretable and not masking heterogeneity among its components, separate multivariable models were fitted for each individual OCT-A metric. Higher PISA was independently associated with lower superficial capillary plexus vessel density (*β* = −0.353, 95% CI −0.620 to −0.085; *p* = 0.0099), lower deep capillary plexus vessel density (*β* = −0.436, 95% CI −0.726 to −0.145; *p* = 0.0034), and larger foveal avascular zone area (*β* = 0.0123 mm^2^, 95% CI 0.0050 to 0.0196; *p* = 0.0010) (Table S2). All three components were individually significant and directionally concordant, confirming that the composite does not obscure divergent patterns across retinal metrics.

Sensitivity analyses supported the robustness of the primary finding (Table S3). The association between higher PISA and worse OCT-A microvascular impairment remained directionally consistent across nested adjustment models, after use of HC3 robust standard errors, after log-transformation of PISA, and after exclusion of participants with severe NPDR/PDR or severely increased albuminuria. In addition, alternative periodontal exposure definitions showed concordant results for BOP%, mean probing depth, and the number of sites with PPD ≥6 mm, whereas tooth loss was not significantly associated with the OCT-A composite.

### 3.3. Secondary Microvascular Outcomes

**Figure 2:**
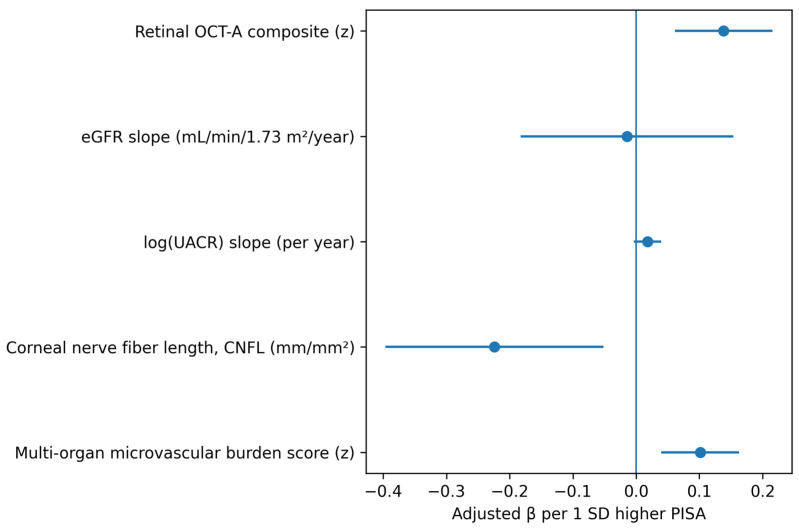
Forest plot of adjusted associations between PISA and primary/secondary outcomes (β per 1 SD higher PISA; whiskers indicate 95% CIs).

Analysis of individual organ-specific secondary outcomes revealed that associations with PISA varied in strength across microvascular beds. PISA was significantly associated with lower CNFL (adjusted *β* = −0.224, 95% CI −0.397 to −0.052; *p* = 0.0113, FDR *q* = 0.023), whereas associations with eGFR slope (*β* = −0.014; *p* = 0.8666) and log(UACR) slope (*β* = 0.018; *p* = 0.1057) were not statistically significant after adjustment. The summary multi-organ microvascular burden score was also associated with PISA (adjusted *β* = 0.101, 95% CI 0.040 to 0.163; *p* = 0.0014, FDR *q* = 0.005), although this composite result should be interpreted in light of the heterogeneity across its individual components. Full model estimates for the primary and secondary outcomes are presented in Table 2 and summarized visually in Figure 2.

### 3.4. Systemic Inflammatory and Endothelial Biomarkers

Higher PISA was associated with a consistent inflammatory and endothelial activation profile (Table 3). After FDR correction (*q* < 0.10), higher PISA was associated with higher hsCRP, IL-6, sICAM-1, sVCAM-1, sE-selectin, PAI-1, angiopoietin-2, and vWF:Ag. Adjusted effect sizes are reported in Table 3, with log-transformed biomarkers expressed as approximate percentage differences per 1-SD increase in PISA.

Key biomarker associations, ordered by FDR *q*-value, included: sICAM-1 (ng/mL; *β* = 26.390; *p* = 0.0000, FDR *q* = 0.0000), IL-6 (pg/mL; approximately 22.4% higher per 1 SD PISA; *p* = 0.0000, FDR *q* = 0.0000), sVCAM-1 (ng/mL; *β* = 67.314; *p* = 0.0000, FDR *q* = 0.0001), hsCRP (mg/L; approximately 18.7% higher per 1 SD PISA; *p* = 0.0001, FDR *q* = 0.0003), sE-selectin (ng/mL; approximately 8.2% higher per 1 SD PISA; *p* = 0.0017, FDR *q* = 0.0027), and PAI-1 (ng/mL; approximately 10.1% higher per 1 SD PISA; *p* = 0.0022, FDR *q* = 0.0030).

## 4. Discussion

In this observational cohort of never-smoking adults with type 2 diabetes, periodontal inflammatory burden quantified by the periodontal inflamed surface area (PISA) was independently associated with quantitative microvascular impairment in the retina and peripheral small fibers, with directionally consistent but non-significant renal trajectory associations. However, the cross-sectional design precludes determination of whether periodontal inflammation contributes to microvascular impairment, whether individuals with more advanced systemic microvascular disease are more susceptible to severe periodontal inflammation, or whether both are driven by shared underlying mechanisms. Higher PISA tracked with worse OCT angiography (OCT-A) perfusion metrics, summarized in a composite score; lower corneal nerve fiber length on corneal confocal microscopy; and a higher multi-organ microvascular burden score; associations with eGFR slope and albuminuria trajectory did not reach statistical significance after adjustment. In parallel, higher PISA was accompanied by a coherent systemic biomarker signature characterized by higher hsCRP and IL-6 and by endothelial activation/injury markers (sICAM-1, sVCAM-1, sE-selectin, PAI-1, and vWF antigen), with Angiopoietin-2 providing an additional mechanistic signal consistent with microvascular dysregulation.

Our retinal findings are consistent with the growing evidence linking periodontitis with diabetic retinopathy (DR), while extending prior work by emphasizing high-resolution, preclinical microvascular endpoints. A recent systematic review and meta-analysis reported a significant association between periodontitis and DR across observational studies [14]. However, most existing studies have relied on dichotomous DR outcomes or broad severity grades that may miss early microvascular damage. OCT-A captures capillary nonperfusion, foveal avascular zone remodeling, and changes in vessel density before clinically apparent DR, and these metrics are increasingly recognized as sensitive correlates of glycemic exposure. For example, OCT-A choriocapillaris flow deficit and related parameters differed by glycemic control even in type 2 diabetes without clinical DR [16], and CGM-derived metrics have also been linked with OCT-A vessel density in diabetes cohorts without retinopathy [25]. Against this background, the independent association observed between PISA and OCT-A microvascular impairment supports the notion that oral inflammatory burden may provide additional explanatory power beyond glucose metrics alone.

While the primary association between PISA and the OCT-A composite was statistically robust (*p* = 0.0005), the effect size warranted careful clinical interpretation. The adjusted *β* of 0.138 indicated that each 1 SD higher PISA was associated with a 0.138 SD worse retinal microvascular impairment score—a modest standardized effect. To place this in context, moving from the 25th to the 75th percentile of PISA (approximately 316 to 827 mm^2^, corresponding to roughly 1.3 SD) would be associated with an approximately 0.18 SD difference in the OCT-A composite, which represents a fraction of the interindividual variability in retinal microvascular status within this cohort. Similarly, the secondary outcome effect sizes (microvascular burden score *β* = 0.101; CNFL *β* = −0.224) are small to modest by conventional benchmarks. These magnitudes are not unexpected for a single modifiable exposure in a multifactorially determined outcome, and are comparable to effect sizes reported for individual cardiometabolic risk factors in relation to early microvascular endpoints in the diabetes literature. Nonetheless, statistical significance should not be conflated with established clinical relevance. Whether these effect sizes translate into meaningful differences in long-term complication risk or whether they are large enough to justify changes in clinical management cannot be determined from cross-sectional data alone and will require prospective studies linking PISA-associated microvascular differences to hard clinical endpoints such as vision loss, progression to clinical nephropathy, or symptomatic neuropathy.

The kidney results align with emerging prospective evidence that periodontal disease is associated with the progression of diabetic kidney disease. In a prospective cohort of patients with poorly controlled type 2 diabetes followed for six months, baseline periodontitis stage and surface-based metrics, including PISA, were associated with a greater decline in eGFR; mediation analysis suggested that the effect was largely independent of improvements in glycemic control over follow-up [17]. Our trajectory-based approach similarly emphasizes renal dynamics rather than cross-sectional staging alone, and the directional consistency with the retinal and neural findings is noteworthy; however, the lack of statistical significance after multivariable adjustment means that an independent association between PISA and renal trajectories cannot be established from the present data, and the renal findings should be considered hypothesis-generating. Additionally, population-level observational data suggest that periodontitis co-occurs with diabetes-related complications more broadly [26]. A recent systematic review synthesizing the evidence on periodontitis and both micro- and macrovascular complications of diabetes reported elevated risks across retinopathy, nephropathy, and neuropathy in patients with periodontitis, further supporting the clinical relevance of integrating periodontal status into multi-organ complication risk assessment [27].

For neuropathy, corneal confocal microscopy (CCM) has matured into a validated approach for detecting early small-fiber loss in diabetes and for predicting incident neuropathy. Longitudinal multicenter data show that CCM metrics, including corneal nerve fiber length, predict the development of diabetic neuropathy [28], and systematic reviews and meta-analyses confirm diagnostic utility in diabetic peripheral neuropathy across diverse cohorts [29]. The association observed here between higher PISA and lower corneal nerve fiber indices is therefore clinically meaningful because it demonstrates an association between an oral inflammatory metric and an objective, early neurodegenerative phenotype that can precede abnormalities on conventional nerve conduction studies. This may help explain why prior studies that used symptom questionnaires or coarse definitions of neuropathy have produced heterogeneous findings.

The systemic biomarker profile provides an additional descriptive layer characterizing the inflammatory and endothelial milieu associated with higher periodontal inflammatory burden. Endothelial activation markers and selectins are increasingly recognized as indicators of the risk of microvascular complications in type 2 diabetes. In a follow-up study, baseline VCAM-1 was associated with incident neuropathy, retinopathy, and nephropathy over approximately 2 years, with selectins contributing to risk stratification by organ complication [20]. In a Swedish prospective cohort, soluble adhesion molecules were investigated as predictors of DR, with evidence supporting a potential role for sE-selectin [30]. These observations are consistent with the broader mechanistic framework of diabetic microangiopathy, in which inflammatory signaling, endothelial activation, and impaired perfusion are recognized as interrelated processes [19]. A recent comprehensive review of vascular endothelial function in periodontal disease has summarized evidence that systemic dissemination of periodontal pathogens and inflammatory mediators can impair endothelial-dependent vasodilation, upregulate adhesion molecule expression, and reduce nitric oxide bioavailability, providing a plausible biological framework through which oral inflammation may be associated with vascular dysfunction [31].

However, the present data cannot determine whether the biomarker elevations observed in association with higher PISA lie on a causal pathway between periodontal inflammation and microvascular outcomes or whether they reflect parallel manifestations of a shared inflammatory susceptibility. Angiopoietin-2 is of particular interest because it has been implicated in endothelial destabilization and vascular permeability; studies in periodontitis with and without diabetes have reported higher Ang-2 levels in more inflamed periodontal states, supporting its selection as a mechanistic add-on biomarker [32]. Importantly, no formal mediation analysis was performed in the present study; therefore, the biomarker associations should be interpreted as descriptive co-associations rather than evidence of mediating pathways. Whether these biomarkers mediate, moderate, or simply co-occur with the observed PISA–microvascular associations remains to be determined through appropriately designed prospective studies incorporating causal mediation frameworks.

The present design addresses common limitations in the periodontitis–diabetes complications literature by pairing a modern periodontal inflammatory metric (PISA) with multi-organ, quantitative microvascular endpoints and CGM-derived glycemic metrics, while restricting the cohort to never-smokers to reduce confounding. Contemporary syntheses emphasize that heterogeneity in periodontal definitions, incomplete adjustment for diabetes severity and cardiometabolic risk, and reliance on coarse complication endpoints have limited inference in many earlier studies [11]. By combining OCT-A microvascular perfusion metrics, renal slope-based outcomes, and CCM-derived small-fiber measures, the study also enables biological triangulation: congruent directionality across three microvascular beds supports a systemic process rather than a purely organ-specific artifact.

A conceptual consideration related to the use of PISA as the primary periodontal exposure warrants discussion. PISA quantifies the surface area of inflamed pocket epithelium by integrating probing depth and clinical attachment level measurements with bleeding on probing (BOP) status [21]. While this makes PISA a biologically grounded continuous measure of periodontal inflammatory burden, the BOP component may not exclusively reflect local periodontal disease severity. In systemically inflamed populations such as individuals with T2DM, gingival bleeding tendency can be amplified by systemic factors including hyperglycemia-induced vascular fragility, platelet dysfunction, and a primed inflammatory state, potentially inflating PISA values independently of local bacterial challenge. Consequently, the observed associations between PISA and microvascular outcomes may partly capture shared systemic inflammatory susceptibility rather than a purely periodontal-to-systemic pathway. The inclusion of alternative periodontal exposures in our sensitivity analyses (BOP%, mean PPD, and number of sites with PPD ≥6 mm) provides some reassurance, as the anatomy-weighted metrics (mean PPD and deep sites)—which are less influenced by systemic inflammatory modulation of bleeding—also showed directionally concordant associations with the OCT-A composite (Table S3). Nonetheless, future studies could consider separating the anatomical and inflammatory components of PISA to clarify the relative contributions of local disease extent versus systemic inflammatory amplification.

The differential strength of associations across individual organ-specific outcomes provides clinically relevant information that composite indices alone cannot convey. The significant associations with retinal vessel density, FAZ area, and corneal nerve fiber length point to specific microvascular beds where periodontal inflammatory burden may have its strongest cross-sectional correlates, whereas the null renal findings suggest either a genuinely weaker relationship, insufficient sensitivity of retrospectively derived trajectory measures, or a longer latency before renal microvascular changes become detectable. We therefore encourage readers to prioritize the individual outcome results when considering clinical implications, and to view the composite scores as complementary summary measures that reduce multiplicity but should not substitute for organ-specific interpretation.

Several limitations should be considered. Despite extensive covariate adjustment, the observational design does not permit causal inference, and the potential for unmeasured confounding deserves particular emphasis. Socioeconomic status, diet quality, physical activity levels, oral hygiene behaviors, and access to dental care were not systematically captured in our study and, therefore, could not be included in the adjusted models. These factors are highly relevant because they may independently influence both periodontal inflammatory burden and microvascular outcomes in T2DM through overlapping biological and behavioral pathways. For example, lower socioeconomic status is associated with poorer glycemic control, less frequent dental attendance, and greater exposure to pro-inflammatory dietary patterns, each of which could inflate the observed PISA–microvascular associations. Similarly, oral hygiene practices directly influence periodontal inflammation and PISA values, yet may also correlate with broader health-related behaviors that affect microvascular risk. While the consistency of our findings across multiple vascular beds and biomarker pathways provides some reassurance that a single unmeasured confounder cannot explain all observed associations, residual confounding cannot be excluded. Future studies should prioritize the systematic collection of socioeconomic indicators, validated dietary and physical activity assessments, and detailed oral hygiene data to enable more comprehensive adjustment and to clarify the extent to which the associations reported here are independent of these important covariates. In particular, reverse causation remains a plausible alternative explanation: individuals with more advanced microvascular disease may exhibit heightened systemic inflammation and immune dysregulation that exacerbates periodontal breakdown, rather than periodontal inflammation driving microvascular injury.

The restriction to never-smokers was a deliberate design choice intended to eliminate smoking as a potent confounder of both periodontal inflammation and microvascular disease; however, this substantially limits the generalizability of our findings. Smoking prevalence remains considerable among adults with T2DM globally, and tobacco exposure independently accelerates endothelial dysfunction, retinal microvascular damage, and peripheral neuropathy through mechanisms that overlap with, and may amplify, those attributed to periodontal inflammation. Consequently, the magnitude and pattern of PISA–microvascular associations observed here may not directly translate to smoking-exposed populations, in whom the relative contribution of oral inflammatory burden could be either attenuated by the dominant vascular effects of smoking or, conversely, amplified through synergistic inflammatory pathways. Future studies should seek to replicate these findings in more representative T2DM cohorts that include current and former smokers, with smoking status incorporated as a key covariate or stratification factor, to clarify whether the associations reported here are generalizable and whether smoking modifies the relationship between periodontal inflammatory burden and microvascular outcomes.

A further consideration concerns the retrospective derivation of renal trajectory outcomes. Although we applied standardized criteria for eGFR slope estimation (requiring at least three measurements spanning at least 12 months) and log-transformed UACR slopes, these data were extracted from routine clinical records and laboratory information systems rather than collected under a prospective research protocol with pre-specified sampling intervals. This introduces potential variability in measurement timing, assay standardization across clinical encounters, and completeness of documentation, which may have attenuated the precision of slope estimates and introduced non-differential measurement error. In contrast, the OCT-A and CCM endpoints were acquired prospectively at T0 under standardized imaging protocols with quality-control thresholds, affording greater measurement consistency. This asymmetry in data acquisition may partly explain why the renal trajectory associations, although directionally consistent with those observed for the retinal and neuropathy endpoints, did not reach statistical significance after multivariable adjustment. Prospective studies with protocol-driven, uniform-interval renal sampling would be better positioned to detect modest but clinically meaningful associations between periodontal inflammatory burden and kidney microvascular trajectories.

Biomarkers were measured at a single time point and reflect circulating levels that may be influenced by acute intercurrent conditions; although strict pre-analytic standardization can mitigate this, residual biological variability remains. Even with a larger planned sample, the ability to detect small interaction effects (e.g., by sex, medication class, or glycemic variability strata) may be limited, and multiple testing across endpoints requires careful control of false discovery. We acknowledge that equal weighting of SCP-VD, DCP-VD, and FAZ area in the OCT-A composite implicitly assumes equivalent biological relevance of each component, which may not fully reflect the pathophysiology of early diabetic retinal microangiopathy. For instance, deep capillary plexus changes may precede superficial plexus alterations in some stages of diabetic microvascular disease, and FAZ enlargement may carry prognostic significance that differs from diffuse capillary dropout. Equal weighting was chosen a priori because the existing literature does not provide a consistent empirical basis for differential weighting, and data-driven approaches (such as principal component analysis) risk overfitting in a single-cohort study without external validation. Importantly, the separate analyses of individual OCT-A components (Table S2) showed directionally concordant and individually significant associations with PISA, indicating that the composite result is not driven by a single component and that the equal-weighting assumption does not obscure divergent component-level signals.

Similar considerations apply to the multi-organ microvascular burden score, which combines standardized metrics from retinal, renal, and neural assessments into a single variable. While this approach has the statistical advantage of capturing cumulative microvascular involvement and reducing multiplicity, it merges biologically heterogeneous systems with distinct pathophysiological timelines and measurement characteristics. A given z-score change in retinal vessel density may not carry the same clinical weight as an equivalent z-score change in eGFR slope or corneal nerve fiber length. We therefore present the multi-organ score as a summary measure of overall microvascular status rather than as a clinically actionable index, and emphasize that the organ-specific results reported separately (Table 2) should guide clinical interpretation. Development of empirically weighted multi-organ composites, informed by prospective outcome data linking individual microvascular measures to hard clinical endpoints, would be a valuable direction for future research.

Clinically, the results support a model in which periodontal inflammatory burden serves as a measurable marker of systemic microvascular vulnerability in type 2 diabetes. It should be noted, however, that the strength of evidence differs across organ systems. The associations with retinal microvascular impairment and corneal nerve fiber loss were statistically robust after full adjustment and FDR correction, whereas the renal trajectory associations were not. The term ‘multi-organ’ in this context therefore reflects directional consistency across vascular beds rather than uniformly significant associations, and future studies should specifically seek to clarify whether periodontal inflammatory burden is independently associated with renal microvascular outcomes.

If confirmed in prospective longitudinal studies that can establish temporality, PISA may complement traditional risk stratifiers by identifying patients who may benefit from intensified microvascular screening, including OCT-A-based assessment of preclinical retinal ischemia and CCM-based screening for early neuropathy. Future work should prioritize replication across independent cohorts, incorporate ethically feasible short-interval follow-up to establish temporality, and evaluate whether periodontal status improves predictive performance beyond established diabetes risk models. Finally, mechanistic studies that integrate periodontal microbiome profiling with endothelial and angiopoietic pathways may clarify whether oral dysbiosis contributes to systemic microvascular injury or whether PISA primarily captures shared inflammatory susceptibility.

## 5. Conclusions

Higher periodontal inflammatory burden (PISA) was independently associated with worse retinal microvascular status measured by OCT-A in never-smoking adults with type 2 diabetes. Periodontal inflammation was also associated with reduced corneal nerve fiber measures, whereas renal trajectory associations were directionally consistent but did not reach statistical significance after adjustment. Accordingly, the evidence for a multi-organ association is strongest for the retinal and neural microvascular beds, with the renal component remaining hypothesis-generating.

These associations were accompanied by a coherent systemic profile of increased inflammation and endothelial activation/injury, reflected by higher hsCRP, IL-6, adhesion molecules/selectins, PAI-1, Ang-2, and vWF:Ag. Collectively, the findings suggest that periodontal inflammatory burden may be associated with microvascular vulnerability in type 2 diabetes; however, longitudinal studies establishing temporality and causality are needed before PISA can be recommended as a component of integrated periodontal–diabetes risk assessment and screening strategies.

The present findings are hypothesis-generating and do not establish causality, temporal directionality, or mechanistic mediation. Prospective validation in independent cohorts, ideally incorporating serial periodontal and microvascular assessments alongside formal mediation analyses, is essential before clinical recommendations can be derived from these associations.

## Figures and Tables

**Table 1:** Baseline characteristics overall and stratified by tertiles of periodontal inflamed surface area (PISA).

Characteristic	Overall (N = 285)	Low PISA (n = 95)	Mid PISA (n = 95)	High PISA (n = 95)	*p*-Value
Age, years	60.8 ± 9.2	59.7 ± 8.6	62.2 ± 9.5	60.2 ± 9.2	0.184
Male, n (%)	150 (52.6%)	47 (49.5%)	49 (51.0%)	54 (57.4%)	0.448
Diabetes duration, years	5.25 ± 4.05	4.81 ± 3.64	5.60 ± 3.92	5.34 ± 4.54	0.387
BMI, kg/m^2^	30.15 ± 4.03	29.21 ± 3.80	30.32 ± 3.68	30.94 ± 4.44	0.013
Systolic BP, mmHg	132.4 ± 13.6	132.2 ± 14.0	132.6 ± 13.4	132.4 ± 13.5	0.983
LDL-C, mmol/L	2.46 ± 0.75	2.57 ± 0.73	2.45 ± 0.75	2.35 ± 0.75	0.121
HbA1c, %	7.11 ± 0.84	7.02 ± 0.86	7.16 ± 0.83	7.15 ± 0.82	0.426
CGM time in range, %	74.7 ± 10.3	75.0 ± 10.3	74.7 ± 10.6	74.4 ± 10.1	0.946
CGM coefficient of variation, %	32.8 ± 7.2	32.7 ± 7.2	32.6 ± 7.1	33.0 ± 7.4	0.938
PISA, mm^2^	560 [316, 827]	244 [156, 312]	560 [466, 636]	1022 [842, 1486]	—
Bleeding on probing, %	30.49 ± 15.64	20.29 ± 9.49	28.19 ± 9.26	43.14 ± 17.18	<0.001
Mean probing depth, mm	3.14 ± 0.43	2.90 ± 0.27	3.07 ± 0.35	3.44 ± 0.45	<0.001
Tooth loss, n	1.57 ± 1.34	1.17 ± 1.05	1.44 ± 1.11	2.11 ± 1.62	<0.001
SGLT2 inhibitor, n (%)	136 (47.7%)	51 (53.7%)	43 (44.8%)	42 (44.7%)	0.358
GLP-1RA, n (%)	99 (34.7%)	37 (38.9%)	33 (34.4%)	29 (30.9%)	0.476
ACEi/ARB, n (%)	199 (69.8%)	66 (69.5%)	71 (74.0%)	62 (66.0%)	0.362
Statin, n (%)	205 (71.9%)	68 (71.6%)	66 (68.8%)	71 (75.5%)	0.719

Data are presented as mean ± SD, median [IQR], or n (%), as appropriate. *p*-values compare characteristics across PISA tertiles using one-way ANOVA for continuous variables and chi-square tests for categorical variables. No *p*-value is reported for PISA because tertiles were defined based on this variable. PISA tertiles were defined by rank-based grouping into three equal-sized groups (n = 95 each).

**Table 2:** Adjusted associations between PISA and microvascular outcomes (β per 1 SD higher PISA).

Outcome	Adjusted β Per 1 SD Higher PISA	95% CI	*p*-Value	Note
Retinal OCT-A composite (z)	0.138	0.061 to 0.216	0.0005	Primary
eGFR slope (mL/min/1.73 m^2^/year)	−0.014	−0.183 to 0.154	0.8666	Secondary (FDR *q* = 0.867)
log(UACR) slope (per year)	0.018	−0.004 to 0.040	0.1057	Secondary (FDR *q* = 0.141)
Corneal nerve fiber length, CNFL (mm/mm^2^)	−0.224	−0.397 to −0.052	0.0113	Secondary (FDR *q* = 0.023)
Multi-organ microvascular burden score (z)	0.101	0.040 to 0.163	0.0014	Secondary (FDR *q* = 0.005)

Values are regression coefficients (*β*) from multivariable linear regression models with each outcome as the dependent variable and PISA (per 1 SD) as the independent variable. All models were adjusted for age, sex, diabetes duration, HbA1c, CGM time in range, CGM coefficient of variation, systolic blood pressure, LDL cholesterol, BMI, SGLT2 inhibitor use, GLP-1 receptor agonist use, ACE inhibitor/ARB use, and statin use. FDR *q*-values were computed using the Benjamini–Hochberg procedure across secondary endpoints (*q* < 0.10 threshold). Abbreviations: CI, confidence interval; CNFL, corneal nerve fiber length; eGFR, estimated glomerular filtration rate; FDR, false discovery rate; OCT-A, optical coherence tomography angiography; PISA, periodontal inflamed surface area; SD, standard deviation; UACR, urinary albumin-to-creatinine ratio.

**Table 3:** Adjusted associations between PISA and systemic biomarkers (β per 1 SD higher PISA; FDR-adjusted q-values shown).

Biomarker	Scale	Adjusted β Per 1 SD Higher PISA	95% CI	*p*-Value	FDR *q*
hsCRP (mg/L)	log-transformed	0.171 (≈18.7% per SD)	0.085 to 0.258 (≈8.8% to 29.4%)	0.0001	0.0003
IL-6 (pg/mL)	log-transformed	0.202 (≈22.4% per SD)	0.115 to 0.289 (≈12.2% to 33.5%)	<0.0001	<0.0001
sICAM-1 (ng/mL)	untransformed	26.390	16.955 to 35.825	<0.0001	<0.0001
sVCAM-1 (ng/mL)	untransformed	67.314	36.986 to 97.641	<0.0001	0.0001
sE-selectin (ng/mL)	log-transformed	0.079 (≈8.2% per SD)	0.030 to 0.128 (≈3.1% to 13.6%)	0.0017	0.0027
PAI-1 (ng/mL)	log-transformed	0.096 (≈10.1% per SD)	0.035 to 0.157 (≈3.6% to 17.1%)	0.0022	0.0030
Ang-2 (ng/mL)	log-transformed	0.067 (≈6.9% per SD)	0.017 to 0.117 (≈1.7% to 12.4%)	0.0096	0.0110
vWF:Ag (%)	untransformed	4.109	0.551 to 7.667	0.0244	0.0244

Values are regression coefficients (*β*) from multivariable linear regression models with each biomarker as the dependent variable and PISA (per 1 SD) as the independent variable. All models were adjusted for age, sex, diabetes duration, HbA1c, CGM time in range, CGM coefficient of variation, systolic blood pressure, LDL cholesterol, BMI, SGLT2 inhibitor use, GLP-1 receptor agonist use, ACE inhibitor/ARB use, and statin use. For log-transformed biomarkers, approximate percentage differences per 1 SD higher PISA are shown in parentheses, computed as (exp(β) − 1) × 100. FDR *q*-values were computed using the Benjamini–Hochberg procedure across all eight biomarkers (*q* < 0.10 threshold). Abbreviations: Ang-2, angiopoietin-2; CI, confidence interval; FDR, false discovery rate; hsCRP, high-sensitivity C-reactive protein; IL-6, interleukin-6; PAI-1, plasminogen activator inhibitor-1; PISA, periodontal inflamed surface area; SD, standard deviation; sE-selectin, soluble E-selectin; sICAM-1, soluble intercellular adhesion molecule-1; sVCAM-1, soluble vascular cell adhesion molecule-1; vWF:Ag, von Willebrand factor antigen.

## Data Availability

The deidentified clinical, periodontal, glycemic, and microvascular data supporting the findings of this study are available from the corresponding author upon reasonable request. Requests will be assessed in accordance with the institutional ethics approval and applicable data protection regulations. Laboratory output files and biomarker records are retained on institutional password-protected systems. No publicly accessible dataset was generated because the data contain participant-level clinical and biomarker information.

## Supplementary Materials

The following supporting information can be downloaded at https://www.mdpi.com/article/10.3390/cimb48060601/s1: Table S1: Periodontitis stage and grade distribution overall and by PISA tertiles; Table S2: Adjusted associations between PISA and individual OCT-A components; Table S3: Sensitivity analyses for the association between periodontal inflammatory burden and the OCT-A microvascular impairment composite.

## Abbreviations

The following abbreviations are used in this manuscript:

ACEi	Angiotensin-converting enzyme inhibitor
ACEi/ARB	Angiotensin-converting enzyme inhibitor/angiotensin receptor blocker
Ang-2	Angiopoietin-2
ANOVA	Analysis of variance
ARB	Angiotensin receptor blocker
BMI	Body mass index
BOP	Bleeding on probing
BP	Blood pressure
CAL	Clinical attachment level
CCM	Corneal confocal microscopy
CGM	Continuous glucose monitoring
CI	Confidence interval
CKD	Chronic kidney disease
CKD-EPI	Chronic Kidney Disease Epidemiology Collaboration
CNBD	Corneal nerve branch density
CNFD	Corneal nerve fiber density
CNFL	Corneal nerve fiber length
CV	Coefficient of variation
DCP-VD	Deep capillary plexus vessel density
DR	Diabetic retinopathy
EDTA	Ethylenediaminetetraacetic acid
eGFR	Estimated glomerular filtration rate
ELISA	Enzyme-linked immunosorbent assay
FAZ	Foveal avascular zone
FDR	False discovery rate
GLP-1	Glucagon-like peptide-1
GLP-1RA	Glucagon-like peptide-1 receptor agonist
HbA1c	Glycated hemoglobin
HC3	Heteroskedasticity-consistent standard error estimator, type 3
HD-OCT	High-definition optical coherence tomography
HRT3-RCM	Heidelberg Retina Tomograph 3 with Rostock Cornea Module
hsCRP	High-sensitivity C-reactive protein
IL-6	Interleukin-6
IQR	Interquartile range
LDL	Low-density lipoprotein
LDL-C	Low-density lipoprotein cholesterol
MDE	Minimum detectable effect
NPDR	Non-proliferative diabetic retinopathy
OCT	Optical coherence tomography
OCT-A	Optical coherence tomography angiography
PAI-1	Plasminogen activator inhibitor-1
PDR	Proliferative diabetic retinopathy
PISA	Periodontal inflamed surface area
PPD	Probing pocket depth
SCP-VD	Superficial capillary plexus vessel density
SD	Standard deviation
SD-OCT	Spectral-domain optical coherence tomography
sE-selectin	Soluble E-selectin
SGLT2	Sodium-glucose cotransporter 2
sICAM-1	Soluble intercellular adhesion molecule-1
sVCAM-1	Soluble vascular cell adhesion molecule-1
T0	Baseline/single-visit study time point
T2DM	Type 2 diabetes mellitus
TIR	Time in range
TLA	Three-letter acronym
UACR	Urinary albumin-to-creatinine ratio
VCAM-1	Vascular cell adhesion molecule-1
vWF:Ag	von Willebrand factor antigen
