# Supplementary material for: Periodontal Inflammatory Burden and Multi-Organ Microvascular Impairment in Type 2 Diabetes: A Cross-Sectional Observational Study
Source: Curr Issues Mol Biol. 2026 Jun 5;48(6):601. doi: 10.3390/cimb48060601 (PMC13297860; doi:10.3390/cimb48060601)
Supplement: Supplementary file 1 [file cimb-48-00601-s001.zip › cimb-4329160-supplementary.pdf]

Supplementary material

Table S1. Periodontitis stage and grade distribution overall and by PISA tertiles.

	Overall (N=285)	Low PISA (n=95)	Mid PISA (n=95)	High PISA (n=95)	*p*-value
Stage					
Stage I	67 (23.5%)	67 (70.5%)	0 (0.0%)	0 (0.0%)	<0.001
Stage II	142 (49.8%)	28 (29.5%)	95 (100.0%)	19 (20.0%)	—
Stage III	60 (21.1%)	0 (0.0%)	0 (0.0%)	60 (63.2%)	—
Stage IV	16 (5.6%)	0 (0.0%)	0 (0.0%)	16 (16.8%)	—
Grade					
Grade A	127 (44.6%)	48 (50.5%)	38 (40.0%)	41 (43.2%)	0.678
Grade B	142 (49.8%)	42 (44.2%)	51 (53.7%)	49 (51.6%)	—
Grade C	16 (5.6%)	5 (5.3%)	6 (6.3%)	5 (5.3%)	—

Data are presented as n (%). *p*-values are computed by comparing the overall distribution across PISA tertiles using chi-square tests. PISA tertiles were defined by rank-based grouping into three equal-sized groups (n=95 each).

Table S2. Adjusted associations between PISA and individual OCT-A components

OCT-A component	Adjusted β per 1 SD higher PISA	95% CI	*p*-value	Direction of worse status
Superficial capillary plexus vessel density, %	-0.353	-0.620 to -0.085	0.0099	Lower = worse
Deep capillary plexus vessel density, %	-0.436	-0.726 to -0.145	0.0034	Lower = worse
Foveal avascular zone area, mm^2	0.0123	0.0050 to 0.0196	0.0010	Higher = worse

Values are regression coefficients from separate multivariable linear regression models with each OCT-A component as the dependent variable and PISA modeled per 1 SD higher value. All models were adjusted for age, sex, diabetes duration, HbA1c, CGM time in range, CGM coefficient of variation, systolic blood pressure, LDL cholesterol, BMI, SGLT2 inhibitor use, GLP-1 receptor agonist use, ACE inhibitor/angiotensin receptor blocker use, and statin use. Negative coefficients for vessel density indicate lower retinal capillary density with higher PISA; positive coefficients for FAZ area indicate larger avascular zone area with higher PISA.

Table S3. Sensitivity analyses for the association between periodontal inflammatory burden and the OCT-A microvascular impairment composite.

Sensitivity analysis	Exposure	N	Adjusted β	95% CI	*p*-value
Primary fully adjusted model	PISA per 1 SD	285	0.138	0.061 to 0.216	0.0005
Model 1: demographic/clinical adjustment	PISA per 1 SD	285	0.199	0.098 to 0.300	0.0001
Model 2: Model 1 + HbA1c	PISA per 1 SD	285	0.167	0.085 to 0.249	0.0001
Model 3: Model 2 + CGM metrics (primary model)	PISA per 1 SD	285	0.138	0.061 to 0.216	0.0005
Fully adjusted model with HC3 robust SEs	PISA per 1 SD	285	0.138	0.054 to 0.222	0.0013
Fully adjusted model, log-transformed exposure	log(1+PISA) per 1 SD	285	0.124	0.045 to 0.202	0.0021
Excluding severe NPDR/PDR	PISA per 1 SD	278	0.139	0.061 to 0.218	0.0006
Excluding severely increased albuminuria	PISA per 1 SD	284	0.139	0.061 to 0.217	0.0005

Alternative exposure model	BOP% per 1 SD	285	0.138	0.061 to 0.214	0.0005
Alternative exposure model	Mean PPD per 1 SD	285	0.087	0.009 to 0.164	0.0289
Alternative exposure model	Sites with PPD ≥6 mm per 1 SD	285	0.092	0.015 to 0.169	0.0190
Alternative exposure model	Tooth loss per 1 SD	285	0.048	-0.033 to 0.129	0.2436

Model 1 included age, sex, diabetes duration, systolic blood pressure, LDL cholesterol, BMI, SGLT2 inhibitor use, GLP-1 receptor agonist use, ACE inhibitor/angiotensin receptor blocker use, and statin use. Model 2 additionally included HbA1c. Model 3 additionally included CGM time-in-range and CGM coefficient of variation and corresponds to the primary fully adjusted model. Robust standard errors were estimated using the HC3 estimator. Alternative exposure models used the same primary adjustment set, with the periodontal exposure substituted as indicated.
